# Intussusception of Heterotopic Gastric Mucosa in the Transverse Colon: A Rare Cause of Perforation and Bleeding

**DOI:** 10.7759/cureus.56142

**Published:** 2024-03-14

**Authors:** Sho Fujiwara, Ryuichi Nishimura, Nozomi Koyamada

**Affiliations:** 1 Department of Surgery, Iwate Prefectural Chubu Hospital, Kitakami, JPN

**Keywords:** cecal perforation, endoscopy findings, submucosal tumor, colonic intussusception, heterotopic gastric mucosa

## Abstract

Heterotopic gastric mucosa in the colorectal region is a rare condition and can be found throughout the gastrointestinal tract. Intussusception in adults is mostly associated with adenocarcinoma and requires prompt surgical intervention, especially in cases of intestinal perforation. Our case report demonstrates a cecal perforation caused by the intussusception of heterotopic gastric mucosa within the transverse colon. The patient presented with substantial hematochezia. Despite the challenges of diagnosing this condition preoperatively and in the ICU, accurate pathologic evaluation is important. The consideration of a heterotopic gastric mucosa is crucial in cases of persistent hematochezia, especially in cases of intussusception. The postoperative course of the patient was characterized by hematochezia, which improved with proton pump inhibitors. The consideration of the possibility of heterotopic gastric mucosa may be a guide to appropriate surgical management and optimization of patient outcomes.

## Introduction

Although heterotopic gastric mucosa can be found in any part of the gastrointestinal tract, the colon is a rare location for it [1-5]. Heterotopic gastric mucosa of the colon has been reported in only 10 cases so far [6]. Intussusception in adults is only 5% of all intussusceptions [7,8]. Meckel's diverticulum commonly contains heterotopic gastric mucosa, which may lead to bleeding [9]. 

Adenocarcinoma is commonly found to cause approximately 33% of adult intussusceptions involving the colon [10]. In addition, it is important to note that intussusception can lead to bowel perforation and tumors may be incidentally discovered during this time. Moreover, if the bowel is perforated, emergent surgery is often necessary [11,12]. Evaluating the tumor can be challenging due to the patient's severe general condition. If the malignant tumor can be resected, lymphadenectomy should be performed while considering the patient's general condition. Therefore, during emergent operations, heterotopic gastric mucosa should be considered as a possible cause of intussusception. However, precise diagnosis remains challenging [11].

Here, we present a case of cecal perforation caused by the intussusception of heterotopic gastric mucosa in the transverse colon of an adult who experienced hematochezia from the anal side of the cecum. Heterotopic gastric mucosa is rare but is important as a differential diagnosis because it can cause intussusception and bleeding in adults. Heterotopic gastric mucosa is a benign tumor, but it can cause bleeding and hematochezia like a malignant tumor. These clinical findings might be helpful in diagnosing this rare tumor and expecting a favorable prognosis, even though patients are in severe conditions like in our case.

## Case presentation

A 44-year-old male patient presented to our hospital with a sudden onset of pain in the right upper quadrant of his abdomen and groin in the winter. He had been experiencing abdominal distention for 10 days and epigastric pain for a few days prior to his visit. The patient had experienced bleeding from the gastrointestinal tract, but there was no follow-up. Upon arrival at our hospital, the patient presented with severe septic shock and diffuse intravascular coagulopathy, as indicated by vital signs and laboratory data.

**Figure 1:**
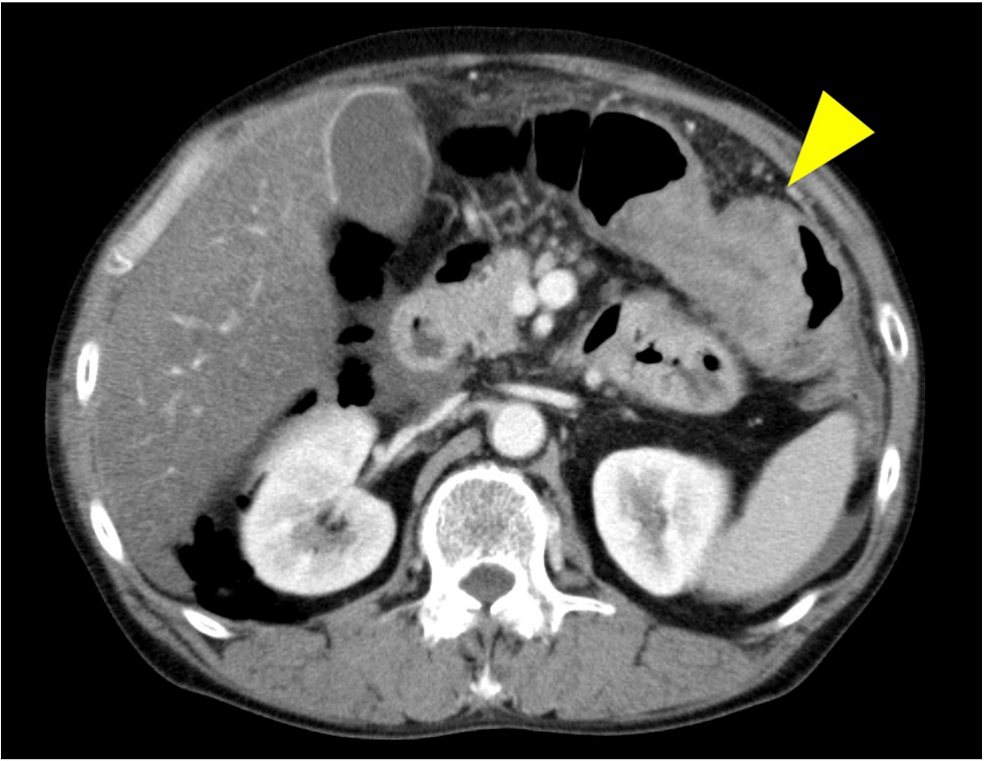
Contrast-enhanced abdominal CT Contrast-enhanced CT revealed perforation of the cecum and the intussusception of a 50 mm tumor in the transverse colon (yellow arrowhead).

Contrast-enhanced CT revealed perforation of the cecum with extensive free air in the retroperitoneal area and intussusception of a 50 mm tumor in the transverse colon (Figure 1). An ileostomy and drainage were performed to remove the perforated necrotic part of the colon. During the operation, platelet count and fibrin degradation products (FDPs) were 20,000/µL and out of the upper measurable range, respectively (Table 1). However, due to the patient's unstable condition and uncontrolled oozing from the dissection area and wound caused by severe septic shock and disseminated intravascular coagulation, we were unable to resect the tumor and observe the tumor enough.

**Table 1:** Laboratory data during the operation Laboratory data suggested severe leukopenia and coagulopathy due to sepsis and disseminated intravascular coagulation. INR, International normalized ratio; APTT, Activated partial thromboplastin clotting time; FDP, Fibrinogen degradation product; CRP, C-reactive protein; LDH, Lactate dehydrogenase; BUN, Blood urea nitrogen; CEA, Carcinoembryonic antigen; CA 19-9, carbohydrate antigen; L, Lower than reference range; H, Higher than reference range; N, Within normal limit

Variables	Values	Comparison with reference range	Reference
White blood cell count (10^3^/uL)	0.93	L	4.0-9.0
Red blood cell count (10^4^/uL)	261	L	400-500
Hemoglobin (g/dL)	8.8	L	13.5-17.5
Hematocrit (%)	24.9	L	40-50
Platelets (10^4^/uL)	2.0	L	13.0-34.0
INR	1.33	H	0.9-1.1
APTT (second)	68.1	H	26.0-38.0
Fibrinogen	98	L	200-400
FDP (ug/mL)	>40	H	<5.0
Lactate (mmol/L)	1.9	H	0.5-1.6
CRP (mg/dL)	9.72	H	<0.41
LDH (IU/l)	341	H	121-226
BUN (mg/dL)	17.4	N	8.0-20.0
Creatinine (mg/dL)	1.21	H	0.5-1.2
Total bilirubin (mg/dL)	1.33	H	0.2-1.2
CEA (ng/mL)	4	N	<5.1
CA 19-9 (U/mL)	30.5	N	<37.1

In the ICU after the operation, we observed hematochezia from the patient's anal side. However, there was no bleeding from the oral side of the stoma, and upper endoscopy during intensive care did not reveal any evidence of bleeding. The hemorrhage was treatable with a proton pump inhibitor (20 mg omeprazole twice a day intravenously) for four days. On postoperative day 42, a colonoscopy was performed to identify the tumor type as we did not have a precise diagnosis. This was due to the potential for a malignant tumor based on the CT. The patient had fully recovered generally from their severe condition in the ICU.

**Figure 2:**
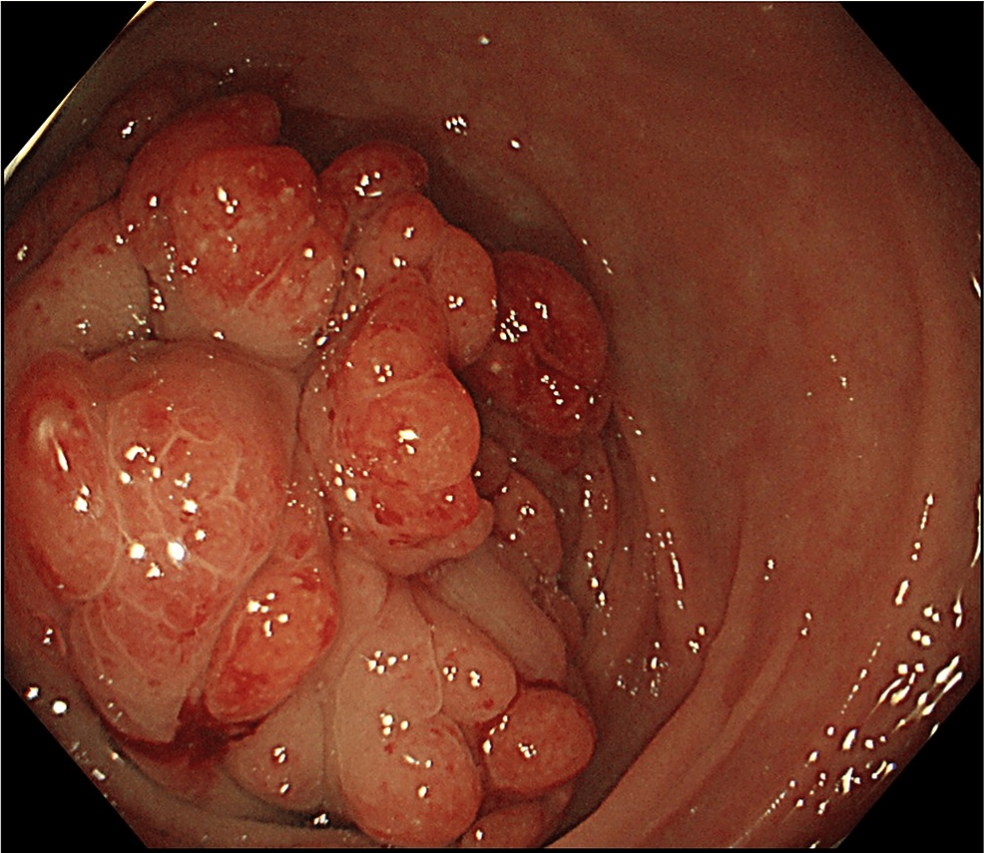
Colonoscopic finding A colonoscopy revealed an approximately 35 mm tumor in the middle of the transverse colon, with multiple nodular changes in the mucosa.

**Figure 3:**
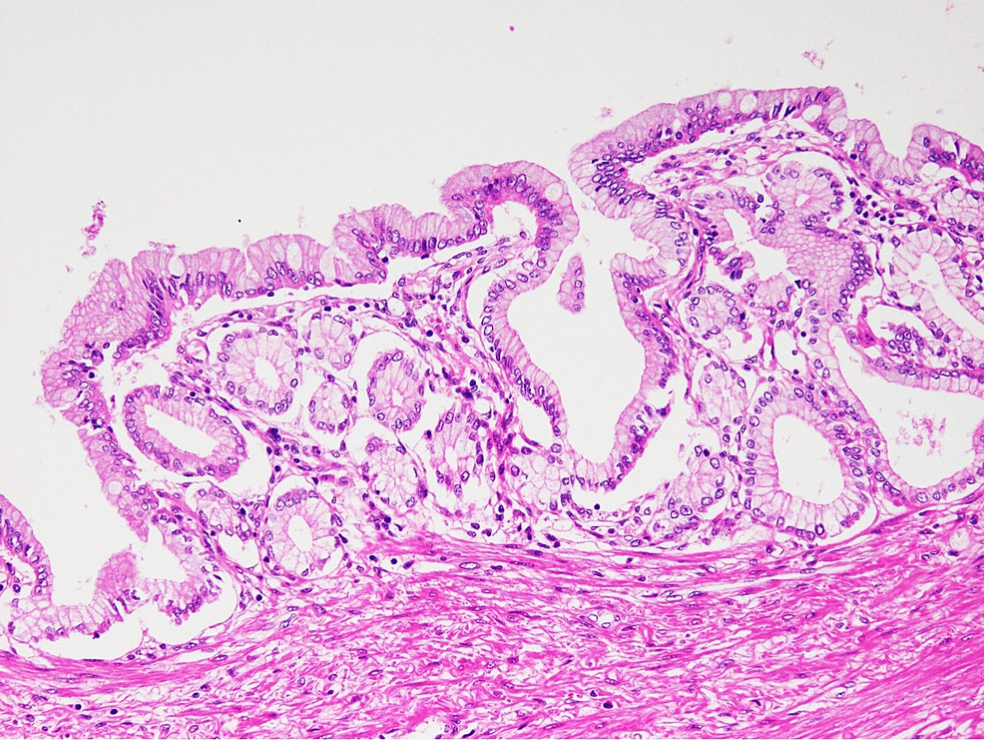
Histology of H&E staining (magnification x80) Histological findings of the resected colon specimen showed a gastric foveolar epithelium.

A colonoscopy revealed an approximately 35 mm tumor in the middle of the transverse colon with multiple nodular mucosal changes (Figure 2). Biopsy results were inconclusive due to excessive mucosa caused by inflammation and surgery. However, these findings suggested that the tumor was a submucosal tumor and not adenocarcinoma. On postoperative day 63, the tumor was removed through transverse colectomy and stoma closure. The surgically resected specimens were histologically analyzed using H&E staining, revealing gastric foveolar epithelium in the colon (Figure 3). The pathological findings of the resected specimens confirmed the diagnosis of heterotopic gastric mucosa. The patient was discharged from the hospital 97 days after the hospitalization and did not require additional nutritional support or medication.

## Discussion

We found two important clinical issues. Diagnosing heterotopic gastric mucosa during emergent surgery and in the ICU can be challenging. Pathological findings are essential for an accurate diagnosis and a biopsy from under the mucosa should be acquired. Additionally, when a tumor causes intussusception and continuous hematochezia, it is important to consider both malignant tumor and heterotopic gastric mucosa until sufficient pathological findings are available.

Heterotopic gastric mucosa is an uncommon occurrence in the transverse colon [1-5]. Intussusception in adults is a rare condition, especially when compared to pediatric cases [6,7]. A previous report from Singapore indicated that only 18.5% of adult intussusception cases occur in the large intestine and that 80% of these cases involve malignancy in the large bowels [10]. Previously reported benign causes of large intestinal intussusception include idiopathic, adenomas, lipomas, hamartomas, hyperplastic polyps, inflammatory polyps, appendiceal inflammation, and cytomegalovirus colitis [13,14]. Although heterotopic gastric mucosa is a rare cause, it can be found anywhere in the gastrointestinal tract. Therefore, when performing emergency surgery for intussusception in adults, potential causes should be considered, including heterotopic gastric mucosa, especially if accompanied by continuous hematochezia. Lymphadenectomy is necessary based on the types of tumors. 

In our case, preoperative CT suggested that the diagnosis was most likely adenocarcinoma, but endoscopic findings after his recovery ruled out adenocarcinoma. He experienced continuous hematochezia before hospitalization and during intensive care. After an ileostomy, bleeding was detected only from the anal side of the stoma, and hematochezia was improved by the use of a proton pump inhibitor. Heterotopic gastric mucosa in Meckel's diverticulum causes hematochezia as in this case [9]. A previous case report suggested that bleeding from heterotopic gastric mucosa was controlled by an H2-antagonist [15]. These findings may be a clue to consider heterotopic gastric mucosa as a differential diagnosis. Technetium-99m pertechnetate scintigraphy is valuable in detecting heterotopic gastric mucosa. However, it is not essential for the diagnosis of all such cases [16,17].

## Conclusions

Although cecal perforation with the intussusception of heterotopic gastric mucosa in the transverse colon is very rare, it should be considered a potential cause of intussusception. Anorectal bleeding improved by a proton pump inhibitor or H2-antagonist use may be a key to the diagnosis of heterotopic gastric mucosa in the colon. Accurate diagnosis of the tumor could optimize surgical procedures.
